# Promoting the Reversible Oxygen Redox Reaction of Li‐Excess Layered Cathode Materials with Surface Vanadium Cation Doping

**DOI:** 10.1002/advs.202003013

**Published:** 2021-01-29

**Authors:** Yongju Lee, Jaewook Shin, Hyeonmuk Kang, Daehee Lee, Tae‐Hee Kim, Young‐Kyun Kwon, EunAe Cho

**Affiliations:** ^1^ Department of Materials Science and Engineering Korea Advanced Institute of Science & Technology Daejeon 34141 Korea; ^2^ Advanced Battery Center KAIST Institute for NanoCentury Korea Advanced Institute of Science and Technology 291 Daehak‐ro, Yuseong‐gu Daejeon 34141 Korea; ^3^ Department of Physics and Research Institute of Basic Sciences Kyung Hee University Seoul 02447 Korea

**Keywords:** cation doping, density functional theory calculation, Li‐excess cathode, lithium‐ion batteries, oxygen redox reaction

## Abstract

Li‐excess layered cathode (LLC) materials have a high theoretical specific capacity of 250 mAh g^−1^ induced by transition metal (cationic) and oxygen (anionic) redox activity. Especially, the oxygen redox reaction related to the activation of the Li_2_MnO_3_ domain plays the crucial role of providing a high specific capacity. However, it also induces an irreversible oxygen release and accelerates the layered‐to‐spinel phase transformation and capacity fading. Here, it is shown that surface doping of vanadium (V^5+^) cations into LLC material suppresses both the irreversible oxygen release and undesirable phase transformation, resulting in the improvement of capacity retention. The V‐doped LLC shows a high discharge capacity of 244.3 ± 0.8 mAh g^−1^ with 92% retention after 100 cycles, whereas LLC delivers 233.6 ± 1.1 mAh g^−1^ with 74% retention. Furthermore, the average discharge voltage of V‐doped LLC drops by only 0.33 V after 100 cycles, while LLC exhibits 0.43 V of average discharge voltage drop. Experimental and theoretical investigations indicate that doped V‐doping increase the transition metal–oxygen (TM—O) covalency and affect the oxidation state of peroxo‐like (O_2_)*^n^* ^−^ species during the delithiation process. The role of V‐doping to make the oxygen redox reversible in LLC materials for high‐energy density Li‐ion batteries is illustrated here.

## Graphical abstract

To improve the cycle retention of lithium‐excess layered cathode (LLC), surface vanadium cation doping is presented. The surface‐doped vanadium cations modify the electronic structure and bonding properties of oxygen anion in the LLC, thereby suppressing the irreversible oxygen extraction and promoting the reversible oxygen redox reaction. Thus, the layered‐to‐spinel‐like phase transformation is mitigated, resulting in the improvement of cycle retention.

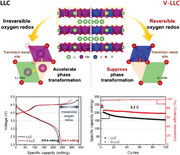

## 1 Introduction

Lithium‐ion batteries (LIBs) have garnered a considerable amount of attention in relation to electronic devices, such as portable devices, electric vehicles, and renewable energy storage systems.^[^ ^1^, ^2^ ^]^ However, current LIBs cannot meet the demanding energy requirements given that the cathode materials used in them, such as LiCoO_2_,^[^ ^3^, ^4^ ^]^ LiFePO_4_,^[^ ^5^, ^6^ ^]^ LiMn_2_O_4_,^[^ ^7^, ^8^ ^]^ and LiMn*_x_*Ni*_y_*Co*_z_*O_2_,^[^ ^9^, ^10^ ^]^ exhibit insufficient specific capacity and still increasable redox potentials. As a high‐energy cathode material, Li‐excess layered cathode (LLC) materials, denoted as (1 − *y*)Li_2_MnO_3_·*y*LiMO_2_ (M = Mn, Co, and Ni, 0 < *y* < 1), are attractive given that their specific capacity exceeds 250 mAh g^−1[^ ^11^, ^12^, ^13^, ^14^, ^15^ ^]^ between 2.0 and 4.8 V.^[^ ^16^, ^17^, ^18^, ^19^ ^]^

The high specific capacity of LLC is related to the oxygen redox reaction (O^2−^/O_2_^2−^ or O^2−^/O_2_ *^n^* ^−^, 3 > *n* > 1) that occurs at potentials above 4.45 V.^[^ ^20^, ^21^ ^]^ However, oxygen redox is irreversible and causes rapid capacity fading.^[^ ^22^, ^23^ ^]^ Specifically, the oxidation of surface oxygen leads to the irreversible evolution of O_2_ gas out of the LLC, leaving oxygen vacancies at the surface and in the sub‐surface region.^[^ ^24^, ^25^ ^]^ These oxygen vacancies facilitate transition metal migration, which induces a phase transformation, resulting in voltage decay during extended cycles.^[^ ^26^ ^]^ Thus, the surface layers of LLC have been modified by coating,^[^ ^27^, ^28^, ^29^ ^]^ doping,^[^ ^30^, ^31^, ^32^ ^]^ and spinel formation^[^ ^33^, ^34^, ^35^ ^]^ strategies to prevent O_2_ evolution and the formation of oxygen vacancies in the surface layers. Meanwhile, as a novel strategy, the use of a reversible oxygen redox reaction has been proposed.^[^ ^36^, ^37^ ^]^ Sathiya et al.^[^ ^20^, ^38^ ^]^ and McCalla et al.^[^ ^39^ ^]^ directly observed the reversible formation of peroxo‐like O—O dimers (≈2.5 Å) in Li_2_RuO_3_ and Li_2_IrO_3_ systems using electron paramagnetic resonance (EPR) and transmission electron microscopy (TEM). Theoretical studies^[^ ^40^, ^41^ ^]^ showed that a strong transition metal—oxygen (TM—O) covalent bond contributes to making the oxygen redox reversible. Yabuuchi et al.^[^ ^42^, ^43^ ^]^ reported a similar reversible oxygen redox in an Nb‐substituted Mn‐based disordered rock‐salt system (Li_1.3_Nb_0.3_Mn_0.4_O_2_), where Nb^5+^ with the *d*° electronic configuration stabilizes the oxygen network and increases the TM—O covalency. These experimental and theoretical studies^[^ ^20^, ^38^, ^39^, ^40^, ^41^, ^42^, ^43^ ^]^ confirmed the applicability of reversible oxygen redox. However, they are based on precious‐metal oxides or disordered rock‐salt systems that exhibit poor electrochemical kinetics during the lithium deintercalation/intercalation process due to the insufficient number of Li‐ion pathways.^[^ ^44^, ^45^ ^]^ Despite commercial importance, the utilization of reversible oxygen redox in conventional layered structures has not yet been reported.

Motivated by the aforementioned considerations, we present vanadium cation (V^5+^) doping in a Mn‐based LLC material to utilize reversible oxygen redox activity by suppressing the irreversible oxygen reaction during the lithium deintercalation/intercalation cycling. Because V^5+^ has a *d*° configuration, similar to Nb^5+^, it is expected to stabilize the oxygen network while enhancing the TM—O covalency in the Mn‐based LLC system. In addition, we propose a novel means of doping V^5+^ cations into the surface layers of LLC, where the processes of irreversible oxygen redox leading to O_2_ evolution and formation of oxygen vacancies are intense. The V‐doped LLC demonstrates superior electrochemical performance in terms of the specific capacity, cycle retention, and rate capability relative to the corresponding outcomes for LLC owing to the reversible oxygen redox reaction at the surface. Furthermore, for the first time to the best of the author's knowledge, we elucidate the oxygen redox mechanism in the V‐doped LLC system in terms of the formation of reversible oxidized O—O dimers and strong TM—O covalent bonding using first‐principles calculations.

## 2 Results and Discussion

**Figure 1:**
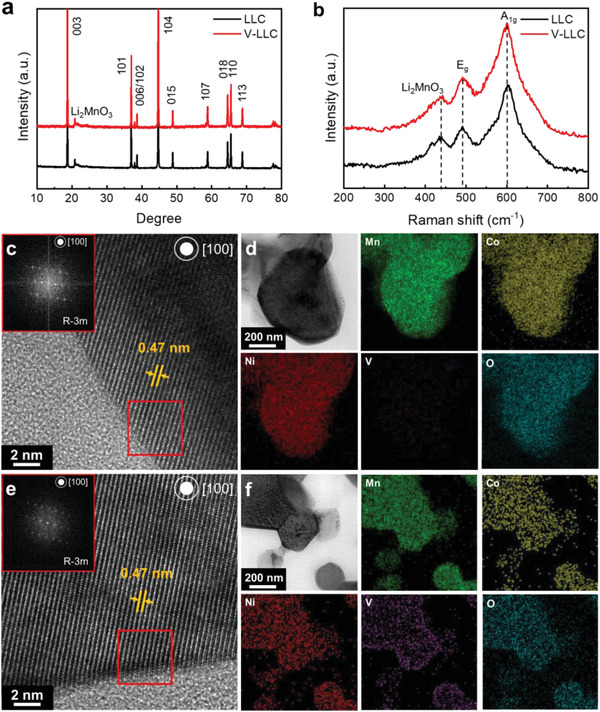
a) XRD patterns and b) Raman spectra of LLC and V‐LLC. c,e) HRTEM images and fast Fourier transform (FFT) images of surface region (red square). The energy dispersive spectrometry (EDS) elemental mappings of d) LLC and f) V‐LLC. (V‐LLC denotes V‐LLC1.)

The LLC, Li_1.2_Mn_0.54_Ni_0.13_Co_0.13_O_2_, is synthesized by a solvothermal reaction and the prepared LLC is surface‐doped with vanadium (V‐doped LLC) using the precursor‐coating method (see the Experimental Section). To find the optimal V‐doping amount, various V compositions of V‐doped LLC samples were prepared by changing ethoxide content in the carbonate precursor‐dispersed solutions from 0.5 to 5 at% with respect to the transition metals in the LLC. Chemical compositions measured by inductive coupled‐plasma technique (ICP‐OES) of the prepared LLC and V‐doped LLC samples are shown in Table S1 in the Supporting Information. With increasing the vanadium ethoxide content, V composition in the prepared V‐doped LLC samples increases from 0.6 to 4.1 at%. The crystal structures of LLC and the V‐doped LLC samples were investigated via the X‐ray diffraction (XRD) patterns and Raman spectra (**Figure** **1**a,b and Figure S3, Supporting Information). For all samples, the main XRD reflections at 18.8°, 37.1°, and 44.7° correspond to the 003, 104, and 101 reflections of the rhombohedral LiMO_2_ phase (M = Mn, Ni, Co; space group R‐3m in its hexagonal setting) (Figure 1a and Figure S3a, Supporting Information). The weak superlattice peaks in the range of 20°–25° are generated from the monoclinic Li_2_MnO_3_ phase (space group C2/m) in the LLC and V‐doped LLC samples. With V contents of 2 (V‐LLC2) and 4 at% (V‐LLC4), additional XRD reflections are observed at 24.3° and 33.2°, corresponding to the Li_3_VO_4_ phase. For quantitative analysis of the lattice parameter, Rietveld refinement was performed (Figure S4, Supporting Information) and the refined lattice parameters were obtained (Table S2, Supporting Information). For all the prepared LLC and V‐doped LLC samples, lattice constants *a* and *c* are found as about 2.85 and 14.23 Å, respectively. These outcomes imply that V‐doping had negligible effects on the lattice parameters. The Raman spectra of LLC and V‐doped LLC samples (Figure 1b and Figure S3b, Supporting Information) exhibit representative vibrational modes of Li‐layered transition metal oxides; the broad bands at 493 and 605 cm^−1^ correspond to the *E*_g_ and *A*_1g_ vibrational modes of the LiMO_2_ phase (R‐3m). A weak and broad vibrational mode at around 400 cm^−1^, corresponding to the Li_2_MnO_3_ phase (C2/m), is also observed in all samples. V‐LLC2 and V‐LLC4 present additional vibration modes of Li_3_VO_4_ at 806.5 and 842.6 cm^−1^, in accordance with the XRD results. These XRD patterns and Raman spectra outcomes demonstrate that the LLC and V‐doped LLC samples have a layered LiMO_2_ structure with the Li_2_MnO_3_ phase and a small amount of V‐doping has negligible effects on the crystal structure of LLC. However, when the V content is 2 at% or more, an impurity phase identified as Li_3_VO_4_ is formed. The electrochemical performances of LLC and V‐doped LLC samples are illustrated in Figure S5 in the Supporting Information. With an increase in the V content up to 1 at%, the electrochemical performances are improved in term of the capacity, cycle retention, and rate capability. However, when the V content is increased to 2 at% and more, the electrochemical performances deteriorate due to the formation of the Li_3_VO_4_ impurity phase, which can negatively affect the electrochemical performance of the V‐doped LLC.^[^ ^46^ ^]^ Based on these results, V‐doped LLC with 1 at% V (V‐LLC1) is investigated in this study. It is henceforth simply denoted as V‐LLC.

The scanning electron microscope (SEM) images demonstrate that LLC and V‐LLC have similar morphologies: spherical secondary particles with a diameter of ≈5 µm and primary particles a few hundreds of nanometers in size (Figure S6, Supporting Information). High‐resolution transmission electron microscopy (HRTEM) images and fast Fourier transform (FFT) images (Figure 1c,e) of the surface region show that both LLC and V‐LLC have identical atomic arrangements with an interlayer distance of 0.47 nm, corresponding to the (003) planes of the R‐3m and the (002) planes of the C/2m structures. These results support the contention that the LiMO_2_ phase (R‐3m) and the Li_2_MnO_3_ phase (C2/m) form in both samples and that V‐doping has negligible effects on the layered structure of the LLC. The elemental mapping images (Figure 1d,f) exhibit that V is mainly doped into the surface of the V‐LLC.

**Figure 2:**
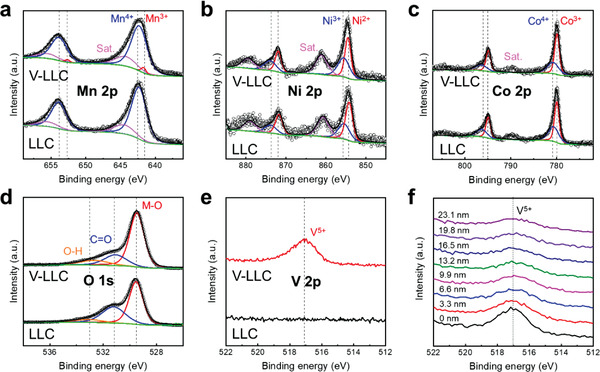
X‐ray photoelectron spectra of a) Mn 2p, b) Ni 2p, c) Co 2p, d) O 1s, and e) V 2p of LLC and V‐LLC and f) depth profile of the V 2p region in V‐LLC. Detection depth is approximated by etching speed and etching time.

X‐ray photoelectron spectroscopy (XPS) was utilized to investigate the oxidation states of LLC and V‐LLC. The survey spectra of LLC and V‐LLC are shown in Figure S7a in the Supporting Information. In accordance with previous studies,^[^ ^47^, ^48^ ^]^ LLC reveals main peaks at 642.6 and 653.8 eV, corresponding to Mn^4+^ 2p_3/2_ and 2p_1/2_, respectively (**Figure** **2**a). V‐LLC presents similar Mn 2p XPS peaks with an additional Mn^3+^ peak at 641.6 eV. Mn 3s X‐ray photoelectron spectra confirm the evolution of Mn^3+^ in V‐LLC (Figure S7b, Supporting Information). The Mn 3s spectra are deconvoluted into two peaks due to the coupling of the nonionized 3s electron with 3d valence‐band electrons.^[^ ^49^ ^]^ The energy difference between the lower and the higher binding energy peaks (Δ*E*_3s_) is considered as a measure of Mn oxidation state.^[^ ^50^ ^]^ Δ*E*_3s_ of LLC and V‐LLC is 4.41 and 4.56 eV, respectively, presenting corresponding average oxidation state of Mn^3.98+^ and Mn^3.82+^. The lower Mn oxidation state of V‐LLC supports evolution of Mn^3+^ in V‐LLC. The Ni 2p spectra of LLC and V‐LLC display Ni^2+^ and Ni^3+^ peaks located at 854.3 and 855.6 eV, respectively (Figure 2b).^[^ ^51^, ^52^ ^]^ The Co 2p spectra of LLC and V‐LLC display Co^3+^ and Co^4+^ peaks located at 780.4 and 781.7 eV (Figure 2c).^[^ ^52^ ^]^ The O 1s spectra of LLC and V‐LLC show peaks at 529.6, 531.7, and 533.0 eV, which are attributed to lattice oxygen (M—O), C=O (Li_2_CO_3_), and O—H (LiOH), respectively (Figure 2d). Li_2_CO_3_ and LiOH can be found on layered oxide cathode surfaces due to the reaction with CO_2_ and moisture during storage.^[^ ^53^ ^]^ Regarding the XPS V 2p region (Figure 2e), V^5+^ is clearly observed only in V‐LLC.^[^ ^46^ ^]^ The depth profile of the XPS V 2p region (Figure 2f) demonstrates that the intensity of the XPS V 2p region decreases with an increase in the detection depth. These results reveal that V^5+^ is successfully doped into the surface layers of V‐LLC. From the Mn 2p, Co 2p, and Ni 2p spectra of LLC and V‐LLC (Figure 2a–c), the proportions of their oxidation states are obtained (Figure S7c, Supporting Information). LLC and V‐LLC have similar ratios of Co^3+^ to Co^4+^ and Ni^2+^ to Ni^3+^. In contrast, while LLC has only Mn^4+^, V‐LLC shows 8% Mn^3+^ and 92% Mn^4+^. These results demonstrate that the oxidation state of V^5+^ leads to the evolution of Mn^3+^ in V‐LLC owing to the charge compensation, whereas V‐doping has negligible effects on the Co and Ni oxidation states.

**Figure 3:**
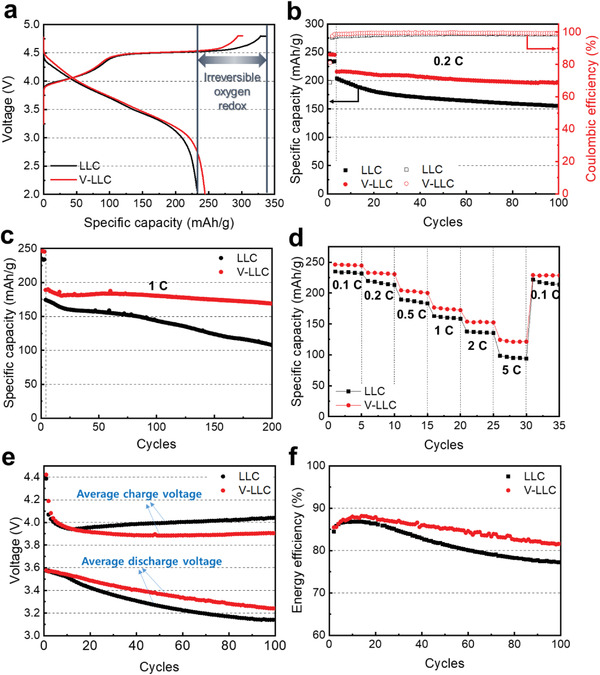
Electrochemical performances of LLC and V‐LLC; a) initial charge–discharge curves at 0.1 C, cycling performances and coulombic efficiencies b) during 100 cycles at 0.2 C and c) during 200 cycles at 1 C after the three activation cycles at 0.1 C, d) rate capability, e) average charge/discharge voltages and f) energy efficiency during 100 cycles at 0.2 C.

The initial charge–discharge curves of LLC and V‐LLC are measured at the current rate of 0.1 C (1 C = 250 mAh g^−1^) in a voltage range from 2 to 4.8 V. **Figure** **3**a shows that the charging behaviors of LLC and V‐LLC are very similar below 4.45 V, implying that the transition metal oxidation reactions occur similarly in the two samples. Above 4.45 V, where oxygen species are oxidized, the LLC delivers a larger capacity than V‐LLC. The corresponding differential capacity versus voltage (d*Q*/d*V*) curves is shown in Figure S8 in the Supporting Information. The first charging process consists of two oxidation reactions: 1) Li^+^ extraction from the layered structure of LiMO_2_ at 4.0 V (O2) with the oxidation reactions of Co^3+^/Co^4+^ and Ni^2+^/Ni^4+^ and 2) partially irreversible Li^+^ extraction^[^ ^54^ ^]^ from the Li_2_MnO_3_ component at 4.5 V (O1) with the anionic oxidation reactions of O^2−^/O_2_ *^n^* ^−^ (1 < *n* < 3).^[^ ^34^ ^]^ The O1 and O2 peaks correspond to the R1 (4.3 V) and R2 (3.7 V) reduction reaction, respectively. The R3 peak at 3.4 V associated with Mn^4+/3+^ reaction.^[^ ^35^, ^55^ ^]^ The O2 peak intensities are almost same for LLC and V‐LLC. In contrast, compared to LLC, V‐LLC exhibits the smaller O1 peak, indicating less oxidation reaction and the smaller charge capacity. Interestingly, V‐LLC demonstrates the larger R1, R2, and R3 peaks than LLC, reflecting more reduction reaction and the larger discharge capacity. As a result, whereas LLC displays a reversible capacity of 233.6 ± 1.1 mAh g^−1^ with an initial coulombic efficiency of 69%, V‐LLC displays a reversible capacity of 244.3 ± 0.8 mAh g^−1^ with excellent initial coulombic efficiency of 81%. The low initial coulombic efficiency of LLC can be related to the irreversible oxygen redox reaction. It is well known that in the Li_2_MnO_3_ component, the oxygen redox reaction occurs irreversibly above 4.45 V.^[^ ^56^ ^]^ These results imply that in V‐LLC, oxygen species are less oxidized than in LLC during the charge process. Nevertheless, V‐LLC demonstrates the higher discharge capacity than LLC, reflecting that the oxygen redox occurs more reversibly. As a result, V‐LLC presents significantly enhanced coulombic efficiency.

**Figure 4:**
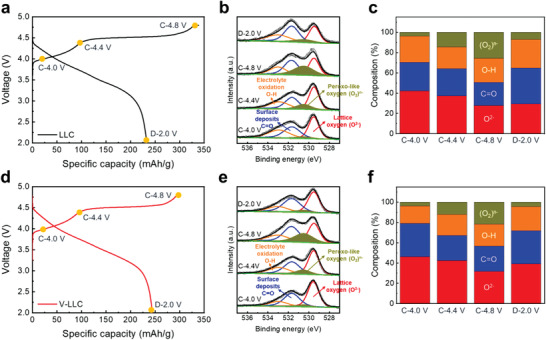
The first cycle voltage profiles of a) LLC and d) V‐LLC measured at 0.2 C, indicating the charge/discharge states for the ex situ XPS measurements. XPS O 1s region measured at C‐4.0, 4.4, and 4.8 V and D‐2.0 V for b) LLC and e) V‐LLC. The peak intensity ratio for various oxygen species of c) LLC and f) V‐LLC.

To investigate the oxygen redox process during first cycle further, ex situ XPS was conducted in the O 1s regions of LLC and V‐LLC at different charge (4.0, 4.4, and 4.8 V) and discharge (2.0 V) states (**Figure** **4**). Compared to the as‐prepared samples, at all states, LLC and V‐LLC exhibit higher intensity of C=O (531.7 eV) and O—H (533.0 eV) peaks, which are associated with the formation of lithium carbonate and oxidation of electrolyte as previously reported.^[^ ^57^, ^58^ ^]^ In both samples, with an increase in charge states from 4.0 to 4.8 V, the characteristic peroxo‐like (O_2_)*^n^* ^−^ species peaks identified at 530.5 eV are intensified, particularly at 4.8 V, implying that O^2−^ species are oxidized to form (O_2_)*^n^* ^−^. In compliance with the evolution of the peroxo‐like (O_2_)*^n^* ^−^ species peaks at higher charge states, the O^2−^ peaks at 529.6 eV decrease in both samples. These results are consistent with the redox activity of oxygen and support the contention that the oxidation of oxide ions occurs during the high‐voltage (>4.45 V) plateau.^[^ ^20^, ^21^ ^]^ Upon discharge to 2 V, although the peroxo‐like peaks disappear in both samples, the O^2−^ peaks show different aspects; the O^2−^ peak in V‐LLC is largely restored as compared to the pristine case (Figure 2d), while the O^2−^ peak in LLC is partially restored. Compositions of those oxygen species are illustrated in Figure 4c,f. These outcomes indicate that the oxygen redox reaction occurs more reversibly in V‐LLC. The reversible oxygen redox reaction contributes to the increases in coulombic efficiency and specific capacity of V‐LLC.

Figure 3b exhibits the discharge specific capacities of LLC and V‐LLC measured at 0.2 C after three activation cycles at 0.1 C. During 100 cycles, the discharge capacity of LLC decreases from 203.6 ± 1.2 to 151.4 ± 1.4 mAh g^−1^, corresponding to a cycle retention of 74% (0.2 C). V‐LLC shows considerably improved cycle performance for 100 cycles. The initial discharge capacity of V‐LLC is 215.8 ± 1.5 mAh g^−1^ and decreases to 196.1 ± 0.9 mAh g^−1^ at the 100th cycle with capacity retention of 92% (0.2 C). The coulombic efficiencies of LLC and V‐LLC after 100 cycles are 98% and 99%, respectively. The cycle stability was evaluated at a high rate (1 C) after three activation cycles at 0.1 C (Figure 3c). After 200 cycles at the high rate, the LLC sample delivers relatively poor cycle retention of 58%, whereas the V‐LLC sample shows a better cycle retention of 88%. Figure 3d presents the rate performance outcomes of LLC and V‐LLC when the current rate ranges from 0.1 to 5 C. The discharge capacities of LLC at 0.1, 0.2, 0.5, 1, 2, and 5 C are 234.8 ± 1.2, 219.8 ± 1.7, 189.8 ± 2.0, 161.4 ± 0.7, 137.7 ± 1.4, and 98.5 ± 0.9 mAh g^−1^, respectively. In contrast, the corresponding capacities of V‐LLC are 246.3 ± 0.9, 232.9 ± 1.5, 204.5 ± 1.4, 176.7 ± 1.1, 153.8 ± 0.8, and 124.2 ± 1.2 mAh g^−1^, respectively. These results demonstrate that V‐LLC can deliver a much higher discharge capacity at a higher current rate compared to LLC. The irreversible oxygen extraction from the surface layers of LLC results in transformation of the layered structure to the spinel‐like structure that has a lower Li ion diffusivity.^[^ ^59^, ^60^ ^]^ Thus, the enhanced discharge capacities at higher current rates imply that the irreversible oxygen oxidation and hence the structural transformation are effectively suppressed by V‐doping. The voltage profiles of LLC and V‐LLC measured at every cycle (voltage profiles at the 1st, 50th, and 100th cycle) are representatively illustrated in Figure S9 in the Supporting Information and the average charge/discharge voltages of LLC and V‐LLC during cycling are obtained as Figure 3e. As illustrated in Figure S9 in the Supporting Information and Figure 3e, during 100 cycles, the average discharge voltage of V‐LLC is decreased by ≈0.33 V, which is less than that of LLC (0.43 V). The decay in the average charge/discharge voltage can be associated with a phase transformation from the layered to the spinel‐like structure.^[^ ^61^ ^]^ Thus, V‐LLC can suppress the phase transformation from the layered to the spinel‐like structure compared to LLC. As a result, V‐LLC (83.1%) delivers higher energy efficiency than LLC (76.4%) at the 100th cycle of 0.2 C, as shown in Figure 3f, where the energy efficiency represents the ratio of the discharge energy to the charge energy of the cathode material.

**Figure 5:**
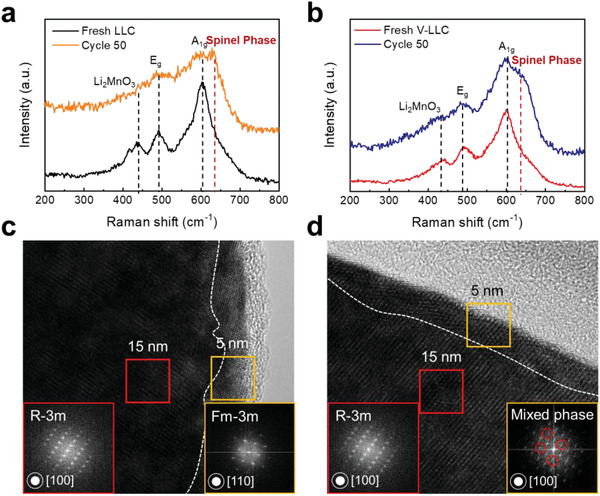
Raman spectra of a) LLC and b) V‐LLC before and after cycling. HRTEM images of c) LLC and d) V‐LLC after cycling; the red and yellow rectangular insets present FFT images of the bulk region and surface region, respectively.

To elucidate the roles of V in improving the electrochemical performances of the LLC material, the phase transformations of LLC and V‐LLC were examined after 50 cycles via the Raman spectra and with HRTEM (**Figure** **5**). As shown in Figure 5a and Figure S10a in the Supporting Information, compared to that of fresh LLC, the Raman spectrum of LLC after 50 cycles clearly reveals that the bands at 400, 493, and 605 cm^−1^, associated with the layered structure, are weakened and that the band at 631 cm^−1^, related to the spinel‐like structure, evolves. These results suggest that the layered structure partially transforms into the spinel‐like structure during cycling (*I*_L_/*I*_S_ = 1.32). On the other hand, V‐LLC demonstrates layered‐structure‐related bands more strongly with a less developed spinel‐related band (*I*_L_/*I*_S_ = 2.09), reflecting that the layered‐to‐spinel phase transformation is considerably alleviated in V‐LLC (Figure 5b and Figure S10b, Supporting Information). The HRTEM images also demonstrate the suppressed layered‐to‐spinel phase transformation in V‐LLC. As shown in Figure 5c,d, the bulk regions of LLC and V‐LLC near ≈15 nm (red box) represent the perfect layered phase (space group R‐3m) according to the atomic arrangements and their corresponding FFT images. In contrast, the surface regions near ≈5 nm (yellow box) show different structures; the surface region of LLC consists of the pure rock‐salt phase (space group Fm‐3m) because a phase transformation takes place, whereas the surface region of V‐LLC consists of a mixed phase (rock‐salt and layered phases). In other words, the surface phase transformation is mitigated in V‐LLC, which can be attributed to the alleviated irreversible lattice oxygen extraction,^[^ ^62^ ^]^ as demonstrated in Figure 3.

**Figure 6:**
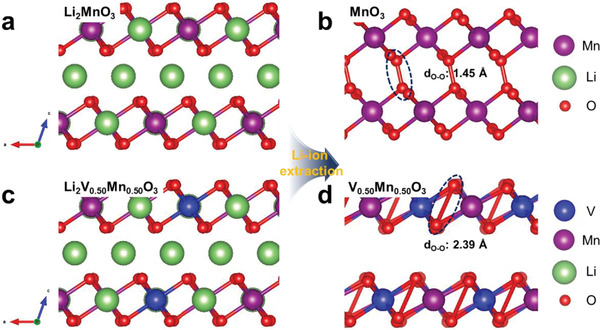
Difference in the structural responses of a,b) Li_2−_ *_x_*MnO_3_ and c,d) Li_2‐_ *_x_*V_0.5_Mn_0.5_O_3_ from *x* = 0 to 2: the formation of different O—O bonds in the MnO_3_ and V_0.5_Mn_0.5_O_3_ determines the irreversible/reversible oxygen redox reaction.

First‐principles calculations are carried out to understand the different oxygen redox reactions of LLC and V‐LLC. Since the oxygen redox reaction occurs in the Li_2_MnO_3_ component, the undoped structure, Li_2−_ *_x_*MnO_3_, and the V‐doped structure, Li_2−_ *_x_*V_0.5_Mn_0.5_O_3_, are examined as model structures. To investigate the discharged and charged environment of the cathode materials, the fully lithiated structures of Li_2_MnO_3_ and Li_2_V_0.5_Mn_0.5_O_3_ and the fully delithiated structures of MnO_3_ and V_0.5_Mn_0.5_O_3_ are constructed. First, the relaxed structures of Li_2−_ *_x_*MnO_3_ and Li_2−_ *_x_*V_0.5_Mn_0.5_O_3_ are illustrated in **Figure** **6**. During the delithiation process, the interlayer distance of the MnO_3_ structure is decreased and the O—O dimer, which has a bond length of 1.45 Å at *x* = 2, is generated (Figure 6b). In contrast, the V_0.5_Mn_0.5_O_3_ structure shows a different structural response. The interlayer distance is well maintained during the delithiation process and the O—O dimer has a greater bond length of 2.39 Å at *x* = 2 (Figure 6d). These different structural responses result in different electronic structures of O—O dimers after delithiation.

**Figure 7:**
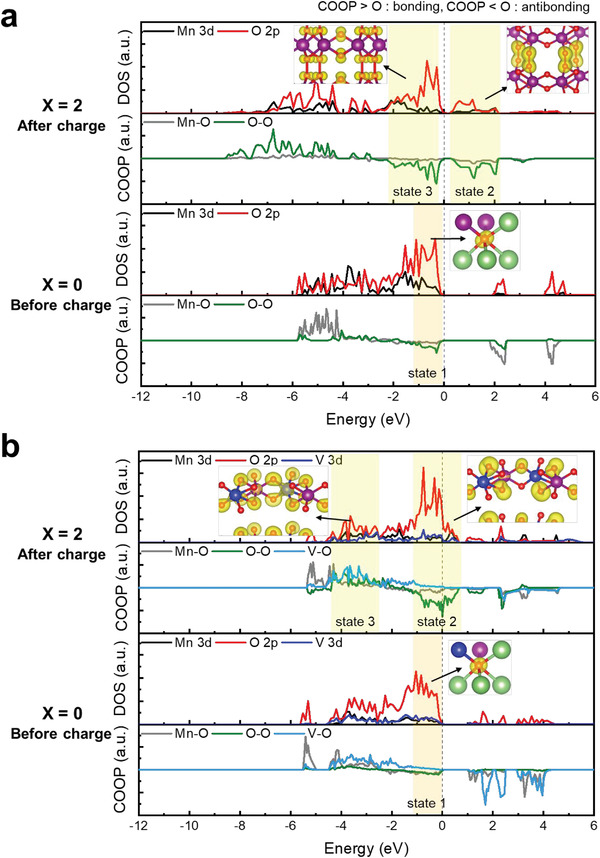
DOS and its corresponding COOP of a) Li_2−_ *_x_*MnO_3_ and b) Li_2−_ *_x_*V_0.5_Mn_0.5_O_2_ structures (*x* = 0: fully lithiated, *x* = 2: fully delithiated). The Mn—O bond, V—O bond, and O—O bond are represented by the gray, sky blue, and green solid lines, respectively. Electron density maps are computed for the selected regions (orange and yellow), which instinctively confirm the existence and type of molecular bonding between the atoms.

The electronic structures of O—O dimers can be studied in terms of density of states (DOS) and crystal orbital overlap populations (COOP). COOP is a powerful bonding descriptor that generates an overlap population weighted DOS.^[^ ^40^ ^]^ In addition to the projected DOS, which provides the relative participation of each atomic level in the electronic band structure of the system, the COOP specify the bonding (COOP > 0), nonbonding (COOP = 0), or antibonding (COOP < 0) characteristic of each electronic state over the system's chemical bonds.^[^ ^63^ ^]^ DOS and corresponding COOP of the TM—O and O—O bonds in the relaxed Li_2−_ *_x_*MnO_3_ and Li_2−_ *_x_*V_0.5_Mn_0.5_O_3_ structures are computed for *x* = 0 and 2, as plotted in **Figure** **7**. For *x* = 0 with Li_2_MnO_3_, the valence band (state 1 in Figure 7a) below the Fermi level is mainly the O 2p band. COOP results present oxygen interacts mostly with Mn (COOP of Mn—O bond > 0), not with oxygen (COOP of O—O bond ≈ 0). The electron density distribution indicates that these electrons are localized in the O 2p orbital lying in the direction of the Li—O—Li bond, resulting in the well‐known orphaned hybridized state.^[^ ^41^, ^64^ ^]^ Upon delithiation (*x* = 2), the formation of the strongly oxidized O—O dimer is identified by the emptying of all of the conduction levels between 0 and 2 eV (state 2 in Figure 7a) and the filling in of the valence level between −2 and 0 eV (state 3 in Figure 7a) with antibonding characteristics (COOP < 0). The electron distribution associated with each level is represented at state 2 and state 3 in Figure 7a. States 2 and 3 are the σ* and π* O—O electronic levels, which correspond to (O_2_)*^n^* ^−^ with *n* ≈ 2 from the O_2_ molecular diagram shown in Figure S11 in the Supporting Information. The structural response of MnO_3_, as mentioned above, is the condensation of the layered structure, characterized by a strongly reduced interlayer spacing arising from the formation of a very short O—O bond (1.45 Å). In the case of Mn—O bonding, the negligible Mn—O COOP obtained for MnO_3_ compared to Li_2_MnO_3_ is compatible with the decreased Mn—O covalency upon delithiation and is consistent with the O—O dimer decoordination from the crystal framework when 2Li are removed.

For *x* = 0 in the Li_2−_ *_x_*V_0.5_Mn_0.5_O_3_ structure, the valence band (state 1 in Figure 7b) below the Fermi level is also mainly the O 2p band, with all interactions being those among Mn, V, and O according to the COOP results. After delithiation (*x* = 2), the O—O COOP intensifies with the appearance of antibonding peaks for V_0.5_Mn_0.5_O_3_ (state 2 in Figure 7b). The electron density illustrated for the antibonding peaks around the Fermi level shows that the O (2p) orbitals are polarized towards the O—O bond direction, e.g., the *σ*‐type orbital. This information clearly indicates the formation of an *σ*‐type O—O bond in V_0.5_Mn_0.5_O_3_, providing direct evidence of electron removal from strongly antibonding σ* electronic states. The observation that one third of the σ* antibonding states are emptied indicates the oxidation state of (O_2_)*^n^* ^−^ with *n* ≈ 3, in full arrangement with the rather long O—O distance (2.39 Å) compared to that of MnO_3_ (1.45 Å). Regarding the TM—O bonding (TM = Mn, V, state 3 in Figure 7b), the clear TM—O COOP with the bonding characteristic remains for V_0.5_Mn_0.5_O_3_ compared to Li_2_V_0.5_Mn_0.5_O_3_, represents strong TM—O covalency during delithiation and indicating the strong coordination of the O—O dimer in the crystal framework when 2Li are removed.

The different oxo (O^2−^) to peroxo‐like (O_2_)*^n^* ^−^ transformations upon delithiation can explain the differences in the initial charge capacities and the reversibility of the discharge capacities of LLC and V‐LLC. From first‐principles calculations, the oxidation of oxygen species generates each peroxo‐like (O_2_)*^n^* ^−^ species with *n* ≈ 2 and *n* ≈ 3 for LLC and V‐LLC, respectively. The formation of irreversible peroxo‐like (O_2_)*^n^* ^−^ species with *n* ≈ 2 indicates more oxygen oxidation, resulting in a larger charge capacity for LLC. In contrast, less oxygen oxidation contributes to less charge capacity but generates more reversible peroxo‐like (O_2_)*^n^* ^−^ species with *n* ≈ 3. Furthermore, more covalent bonding induces a large reversible discharge capacity of V‐LLC and inhibits the decoordination of (O_2_)*^n^* ^−^ species in the crystal framework. As mentioned above, the absence of (O_2_)*^n^* ^−^ species in the crystal framework affects the TM migration, resulting in a layered‐to‐spinel transformation. Accordingly, the increased TM—O covalent bonding in the V‐LLC is responsible for the structural stability with fewer layered‐to‐spinel transformations.

## 3 Conclusion

In this study, we improve the electrochemical performances of an LLC material (Li_1.2_Mn_0.54_Ni_0.13_Co_0.13_O_2_) through surface doping with the V^5+^ cation, which has the *d*° configuration. V is selectively doped onto the surfaces of the primary particles of LLC through a precursor coating method. The prepared V‐doped LLC (V‐LLC) suppresses the irreversible oxygen oxidation and shows more stable cycle retention and rate performance outcomes than LLC. V‐LLC also shows enhanced energy efficiency due to the stable average charge/discharge voltage compared to that of LLC. Ex situ XPS demonstrates the reversible oxygen redox reaction of V‐LLC during the first charge/discharge process, and HRTEM and Raman spectroscopy analyses indicate the detailed structural evolution after cycling, demonstrating that V‐doping can alleviate the undesired phase transformation into detrimental the spinel‐like and rock‐salt phase, which is preferentially initiated from the surface oxygen loss. This work demonstrates the origin of different oxygen redox reactions through DOS and COOP analyses as descriptors of reversible versus irreversible redox activity. V‐doping leads to peroxo‐like (O_2_)*^n^* ^−^ species with *n* ≈ 3 and a strong TM—O covalent bonding condition for the reversible anionic redox. Consequently, we believe this work redefines V‐doping as a viable solution for various cathode materials associated with surface oxygen redox issues and contributes to future advanced designs of high‐energy density cathode materials.

## 4 Experimental Section

##### LLC and V‐Doped LLC Synthesis

The LLC material, Li_1.2_Mn_0.54_Ni_0.13_Co_0.13_O_2_, was synthesized using a solvothermal method followed by a heat treatment. Manganese acetate tetrahydrate (Mn(CH_3_COO)·4H_2_O), nickel acetate tetrahydrate (Ni(CH_3_COO)·4H_2_O), cobalt acetate tetrahydrate (Co(CH_3_COO)·4H_2_O), lithium hydroxide monohydrate (LiOH·H_2_O), and urea (CH_4_N_2_O) were used as precursors. 3.308 g of Mn(CH_3_COO)·4H_2_O, 0.808 g of Ni(CH_3_COO)·4H_2_O, 0.809 g of Co(CH_3_COO)·4H_2_O (stoichiometric ratio of Mn:Ni:Co = 0.54:0.13:0.13), and 4.504 g of urea were dissolved in 50 mL of deionized (DI) water with 25 mL of ethanol as a solvent under continuous stirring for 30 min. The prepared solution was moved into an 80 mL Teflon‐lined autoclave. The autoclave was then kept in an oven at 200 °C for 10 h. After cooling to room temperature, the resultant was filtered and washed with ethanol and DI water at least three times and dried at 80 °C in a vacuum oven overnight to obtain the carbonate precursor. The prepared carbonate precursor and LiOH·H_2_O (5% excess) were mixed, pressed into a pellet, calcined in a furnace at 500 °C for 6 h, and subsequently heat‐treated at 900 °C for 12 h. Heating rate was 5 °C min^−1^. The pellet was then naturally cooled to room temperature and ground to obtain the LLC material.

The surface of the LLC material was doped with vanadium (V) using a precursor coating method (Figure S1, Supporting Information). First, the prepared carbonate precursor was dispersed in ethanol containing 0.5, 1, 2, and 5 at% vanadium ethoxide; atomic ratio of V to transition metals in the LLC was 0.5, 1, 2, and 5 at%. The carbonate precursor‐dispersed solutions were kept at 80 °C under constant stirring until the ethanol evaporated. The dry powder was collected and calcined with LiOH·H_2_O under a condition identical to that used with the pristine LLC.

##### Electrochemical Measurements

The electrochemical properties of the prepared samples were measured using a 2032‐type coin cell. The slurry was prepared by mixing 80 wt% of the active material (Li_1.2_Mn_0.54_Ni_0.13_Co_0.13_O_2_), 10 wt% of conductive super P carbon, and 10 wt% of polyvinylidene fluoride (PVDF) as a binder in N‐methyl‐2‐pyrrolidinone (NMP). The prepared slurry was cast onto an aluminum (Al) foil current collector using the doctor‐blade method and was dried at 80 °C overnight in a vacuum oven. The loading mass of the active material was ≈4–5 mg cm^−2^. The electrolyte was prepared by mixing 1 m LiPF_6_ in ethylene carbonate (EC) and 1 m LiPF_6_ in a diethyl carbonate (DEC) solution at a 1:1 volume ratio. Electrochemical cycling tests of the prepared cells were performed in a voltage range of 2.0–4.8 V (vs Li^+^/Li). To confirm reproducibility of the electrochemical performance, four samples of LLC and V‐doped LLCs were tested, respectively, under a given condition.

##### Materials Characterization

The crystalline phases of the LLC and V‐LLC materials were identified by XRD (Rigaku SmartLab) with a powder X‐ray diffractometer using Cu K_*α*_ (*λ* = 1.5418 Å) radiation. An elemental analysis of the synthesized materials was carried out using the ICP‐OES (Agilent ICP‐OES 5110). The morphology of the materials was observed with an SEM (Phillips XL30) and a HRTEM (JEM‐ARM200F) equipped with an energy dispersive spectrometry (EDS) detector. A Raman spectroscopy (LabRAM HR Evolution, HORIBA Jobin Yvon) assessment was conducted using a 514 nm laser. The cycled electrodes were analyzed after 50 cycles with fully lithiated state to identify the structural change after electrochemical cycling. To prepare the cycled electrode samples for the Raman spectroscopy analysis, the electrodes were sealed under argon between two slide glasses with Kapton tape in a glove box. XPS (K‐Alpha+ Thermo Fisher Scientific) was then used to determine the ion valence states in the cathode materials. From the energy difference between the lower and the higher binding energy peaks (Δ*E*_3s_) of Mn 3s X‐ray photoelectron spectra, the average Mn oxidation state (AOS) can be estimated using the equation; AOS = 8.956 − 1.126Δ*E*_3s_.^[^ ^48^, ^50^ ^]^ The depth profiles were obtained with etching of the surface by an Ar ion beam at an etching rate of ≈0.33 nm s^−1^. To conduct XPS analyses for the charge and discharge states of the cathode materials, the coin cells were charged to 4.0, 4.4, and 4.8 V and discharged to 2.0 V and disassembled. Then, the cathode materials were collected and loaded onto a sample holder in a glove box. Subsequently, the sample holder was made vacuum state in a vacuum chamber and transferred to the XPS chamber without exposure of the cathode materials to the air.

##### Computational Method

First‐principles calculations were performed based on density functional theory (DFT) as implemented in the Vienna ab initio simulation package (VASP)^[^ ^65^ ^]^ with the projector augmented‐wave method to describe the valence electrons. The exchange‐correlation functional was employed with spin‐polarized generalized gradient approximation (GGA) with the Perdew–Burke–Ernzerhof method.^[^ ^66^ ^]^ To describe the onsite Coulomb interaction of the 3d state of Mn and V, Hubbard U correction^[^ ^67^ ^]^ was used with the *U*_eff_ parameter taken from a previous report (*U*_eff_ = 5.0, 3.0).^[^ ^68^ ^]^ The antiferromagnetic (AF) arrangements of the spin on the Mn ions were established in a previous theoretical report by Singh.^[^ ^69^ ^]^ Supercell structures were generated with a four‐unit cell for Li_2_MnO_3_ and then replaced half of the Mn atoms with V atoms in a supercell to form Li_2_V_0.5_Mn_0.5_O_3_, as illustrated in Figure S2a,c in the Supporting Information. The crystal orbital overlap population (COOP) was calculated using the Lobster program developed by Dronskowski et al.^[^ ^63^, ^70^, ^71^ ^]^

## Conflict of Interest

The authors declare no conflict of interest.

## Supporting information

Supporting Information
