# Supplementary material for: Promoting the Reversible Oxygen Redox Reaction of Li‐Excess Layered Cathode Materials with Surface Vanadium Cation Doping
Source: Adv Sci (Weinh). 2021 Jan 29;8(6):2003013. doi: 10.1002/advs.202003013 (PMC7967087; doi:10.1002/advs.202003013)
Supplement: Supplementary file 1 — Supporting Information [file ADVS-8-2003013-s001.pdf]

© 2020 Wiley-VCH GmbH

ADVANCED SCIENCE Open Access

Supporting Information

for *Adv. Sci.*, DOI: 10.1002/advs.202003013

Promoting the Reversible Oxygen Redox Reaction of Li-excess Layered Cathode Materials with Surface Vanadium Cation Doping

*Yongju Lee[a], Jaewook Shin[a,b,], Hyeonmuk Kang[a], Daehee Lee[a], Tae-Hee Kim[a],Young-Kyun Kwon[c], EunAe Cho[*a,b]*

Supporting Information

Promoting the Reversible Oxygen Redox Reaction of Li-excess Layered Cathode Materials with Surface Vanadium Cation Doping

Yongju Lee[a], Jaewook Shin[a,b,], Hyeonmuk Kang[a], Daehee Lee[a], Tae-Hee Kim[a],Young-Kyun Kwon[c], EunAe Cho[*a,b]

[a] ***Department of Materials Science and Engineering, Korea Advanced Institute of Science & Technology, Daejeon, Korea***

[b] ***Advanced Battery Center, KAIST Institute for NanoCentury, Korea Advanced Institute of Science and Technology, 291 Daehak-ro, Yuseong-gu, Daejeon, 34141, Republic of Korea***

[c] ***Department of Physics and Research Institute of Basic Sciences, Kyung Hee University, Seoul, Korea***

*Corresponding authors Tel.: +82-42-350-3317

Email addresses: eacho@kaist.ac.kr

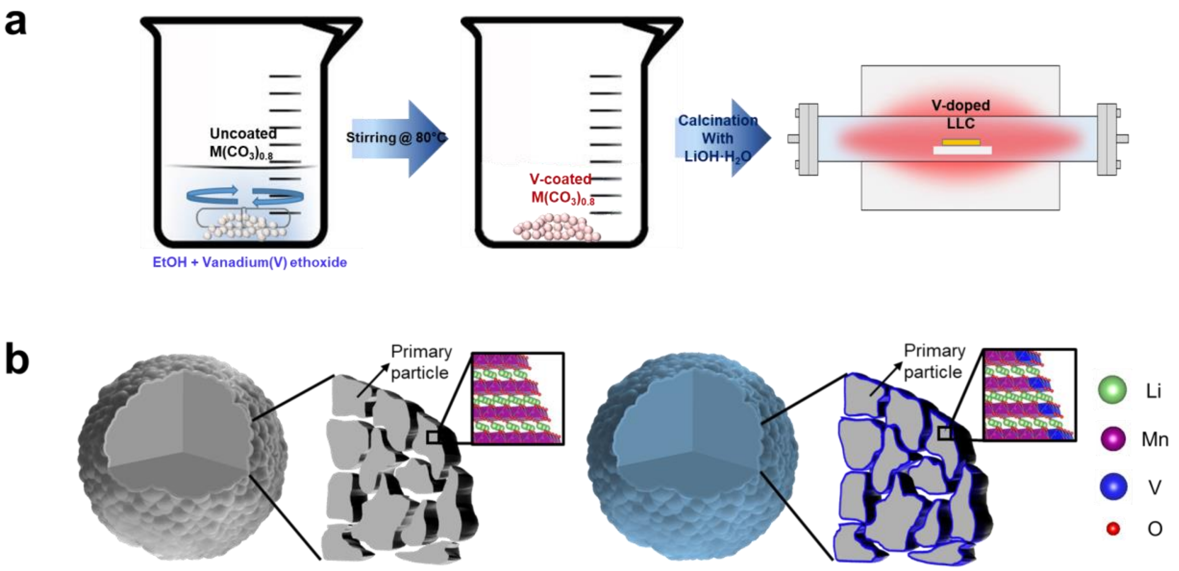

Figure S1. (a) A schematic illustration of the precursor coating method to prepare V-doped LLC. (b) Schematic representations of the LLC (left) and V-doped LLC (right) particle; the V cations (colored in blue) are mainly doped on the surface of primary particles by using the precursor coating method. (Inset: crystal structures of LLC (left) and V-doped LLC (right)).

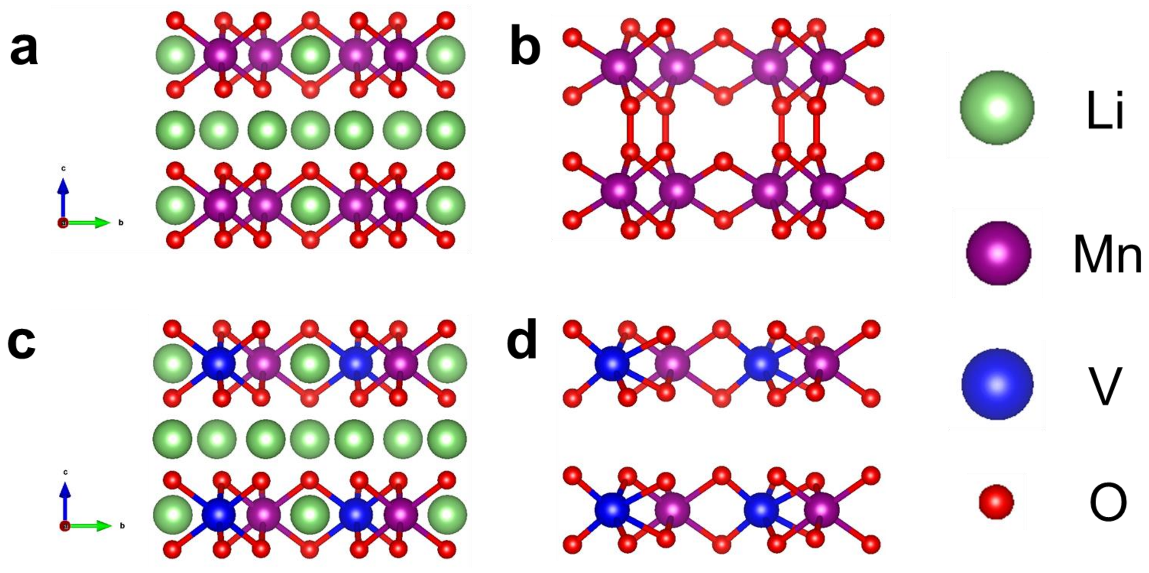

Figure S2. Optimized layered structures of (a) fully lithiated Li_2MnO_3, (b) fully delithiated MnO_3, (c) fully lithiated $Li_2V_{0.5}Mn_{0.5}O_3$, and (d) fully delithiated $V_{0.5}Mn_{0.5}O_3$.

Table S1. Chemical compositions of LLC and V-doped LLCs with various V ratios measured by ICP-OES.

Vadium ethoxide content (at%)	Measured atomic ratio				Notation
	Mn	Ni	Co	V	
**0**	0.543	0.133	0.131	-	LLC
**0.5**	0.534	0.136	0.132	0.006	V-LLC0.6
**1.0**	0.539	0.130	0.128	0.010	V-LLC1 (V-LLC)
**2.0**	0.538	0.132	0.134	0.021	V-LLC2
**5.0**	0.541	0.134	0.131	0.041	V-LLC4

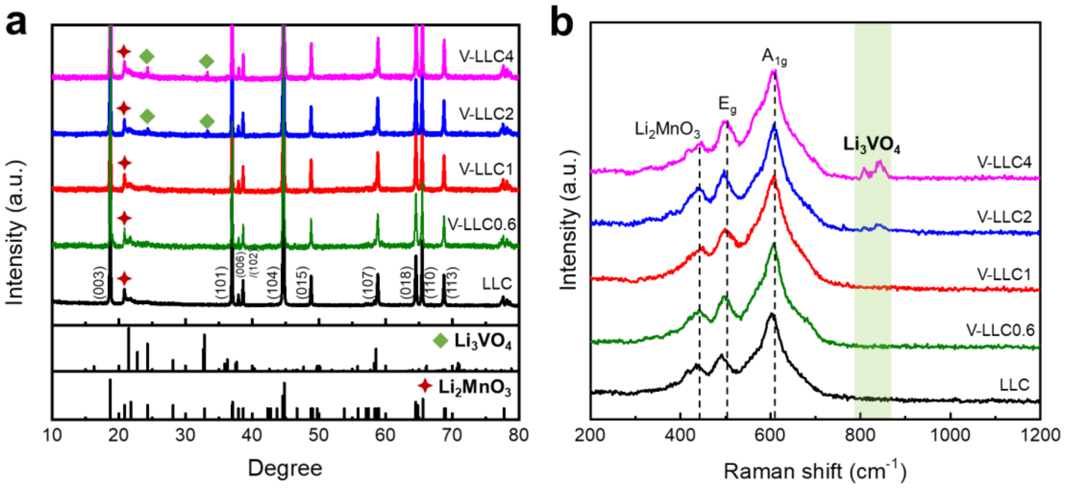

Figure S3. (a) XRD patterns and (b) Raman spectra of LLC and V-LLCs.

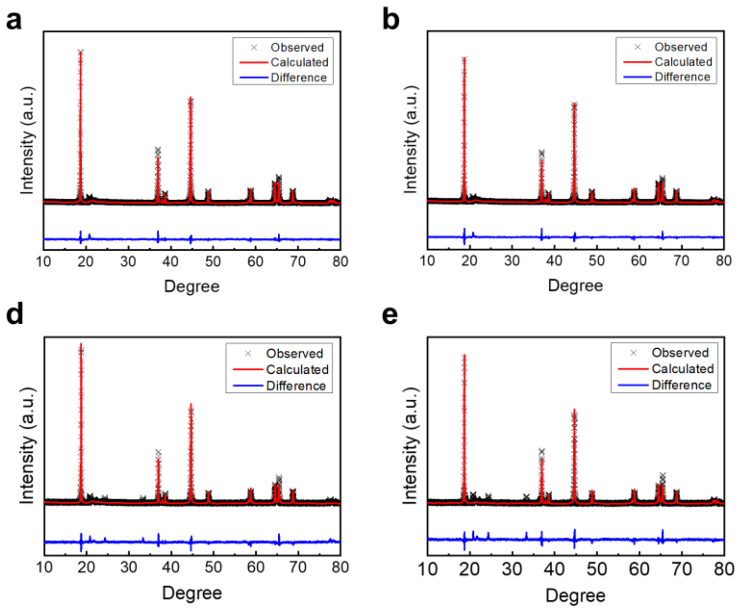

Figure S4. Rietveld refinement for the XRD patterns of (a) LLC, (b) V-LLC0.6, (c) V-LLC1, (d) V-LLC2 and (e) V-LLC4.

Table S2. Refined lattice parameters of LLC and V-LLCs.

**Materials**	**a (Å)**	**c (Å)**	**c/a ratio**	**Refined parameter**	
				R_p	R_{wp}
**LLC**	2.849	14.231	4.995	4.84	5.85
**V-LLC0.6**	2.848	14.232	4.997	4.35	5.12
**V-LLC1 (V-LLC)**	2.850	14.233	4.994	4.71	5.90
**V-LLC2**	2.847	14.234	4.998	5.65	7.44
**V-LLC4**	2.848	14.231	4.996	5.83	7.94

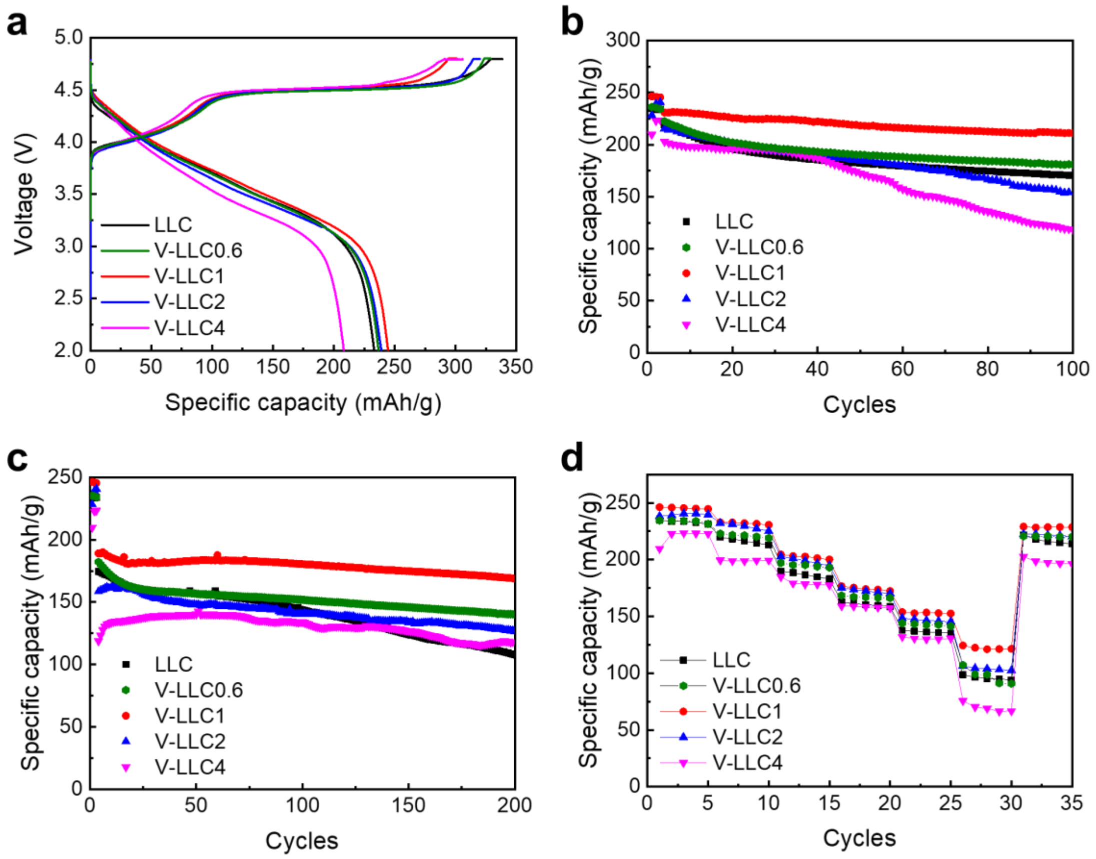

Figure S5. Electrochemical performances of LLC and V-doped LLCs; (a) the first cycle charge-discharge curves, cycling performances at (b) 0.1 C and (c) 1 C rate and (d) rate capability outcomes.

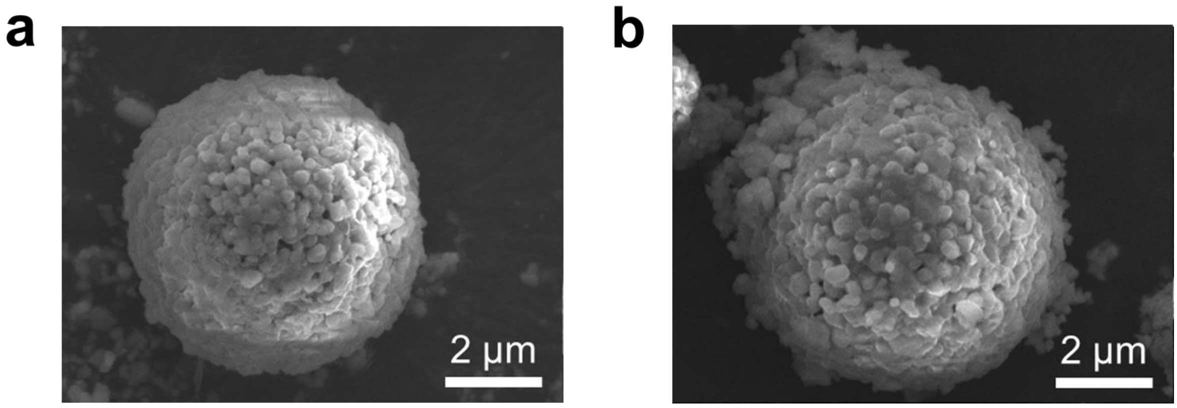

Figure S6. SEM images of (a) LLC and (b) V-LLC.

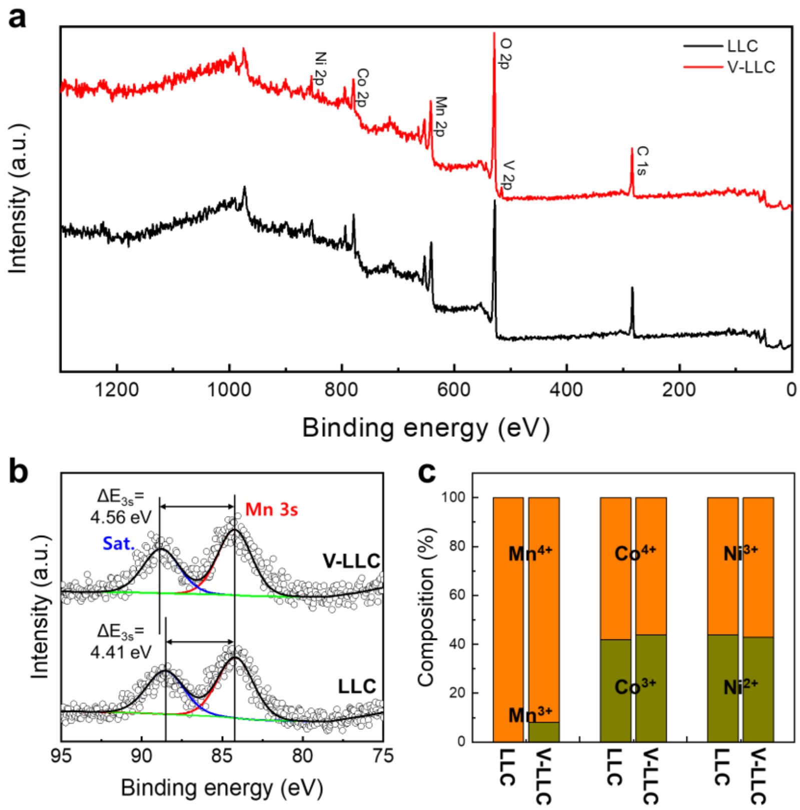

Figure S7. (a) X-ray photoelectron survey spectra, (b) Mn 3s XPS region (red and blue peaks represent Mn 3s and satellite, respectively.) and (c) the peak intensity ratio for the oxidation states of transition metal components of LLC and V-LLC.

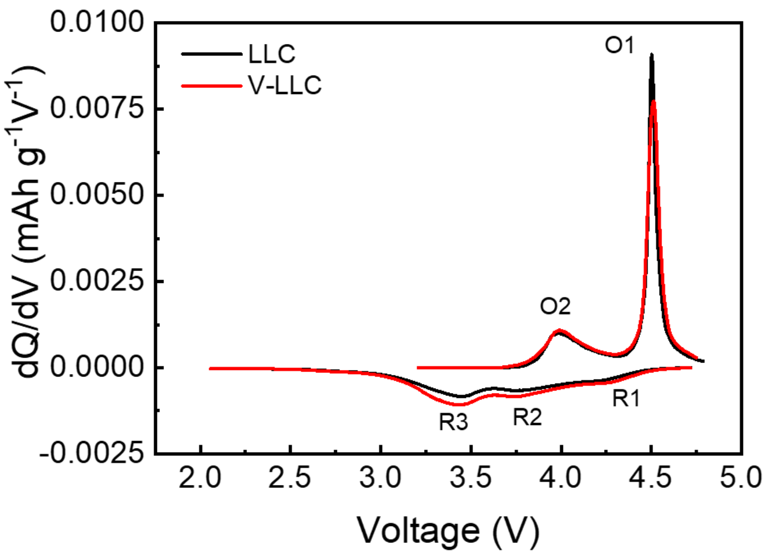

Figure S8. Differential capacity (dQ/dV) curves for the first cycle of LLC and V-LLC.

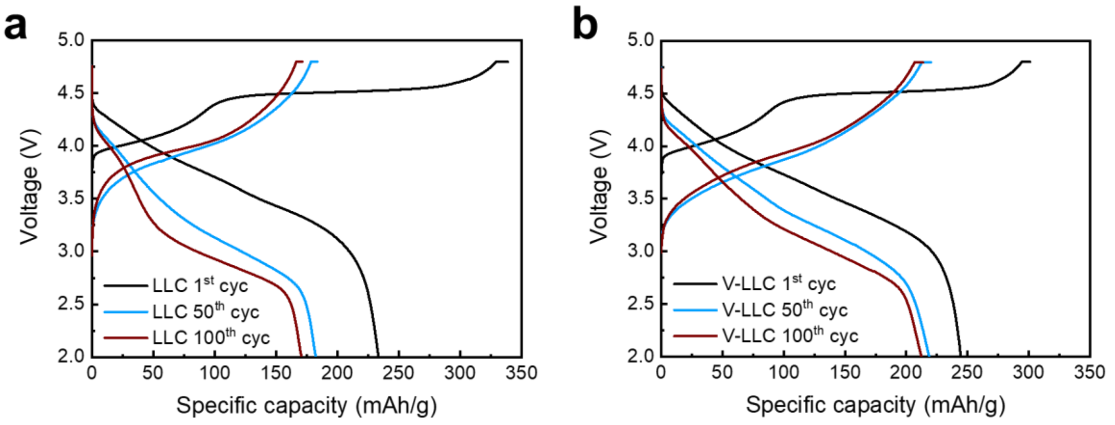

Figure S9. Charge-discharge curves at the 1st, 50th and 100th cycle of (a) the LLC and (b) the V-LLC samples (1st cycle : 0.1 C, 50th and 100th cycle : 0.2 C).

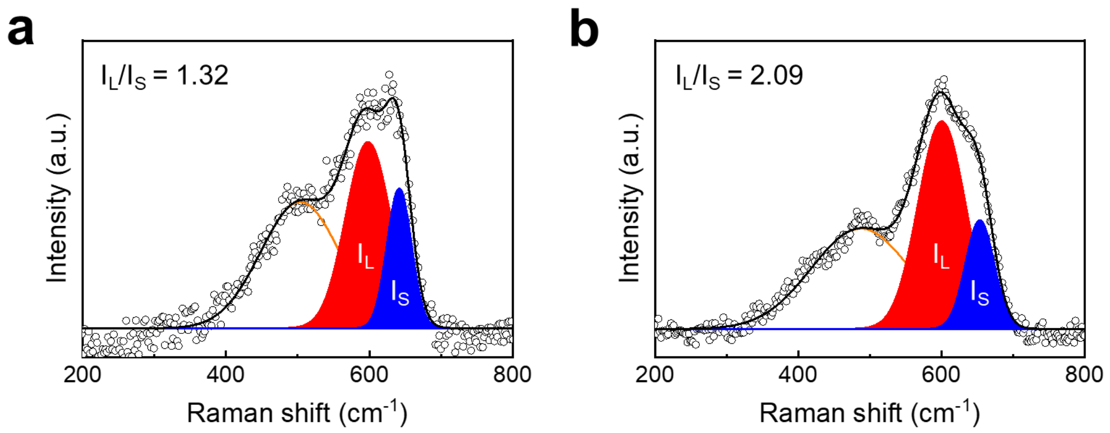

Figure S10. Raman spectra and fitted results of (a) LLC and (b) V-LLC after 50 cycles. I_L (red) and I_S (blue) indicate intensities of the layered structure and the spinel structure, respectively. The higher I_L-to-I_S ratio (I_L/I_S) imply the higher proportion of the layered structure to the spinel structure.

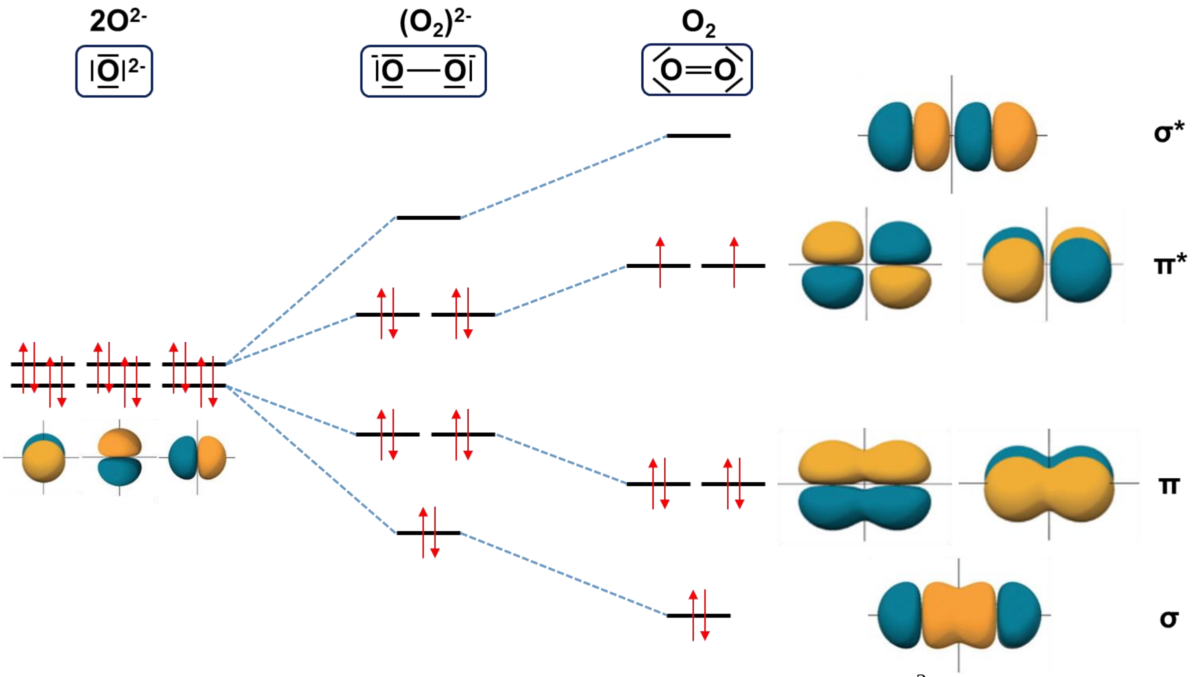

Figure S11. Orbital interaction diagram for the formation of the $(O_2)^{2-}$ peroxide and O_2 gas from the progressive oxidation of two non-interacting O^{2-} anions. The shortening of the O-O distance leads to the splitting of the O^{2-} orbitals into two σ- and four π-type molecular orbitals.
